# Synthesis, mol­ecular and crystal structures of 4-amino-3,5-di­fluoro­benzo­nitrile, ethyl 4-amino-3,5-di­fluoro­benzoate, and diethyl 4,4′-(diazene-1,2-di­yl)bis­(3,5-di­fluoro­benzoate)

**DOI:** 10.1107/S2056989024006819

**Published:** 2024-07-19

**Authors:** Egor M. Novikov, Jesus Guillen Campos, Javier Read de Alaniz, Marina S. Fonari, Tatiana V. Timofeeva

**Affiliations:** a Department of Chemistry New Mexico Highlands University,Las Vegas New Mexico 87701 USA; b Department of Chemistry and Biochemistry University of California Santa Barbara Santa Barbara CA 93106 USA; c Institute of Applied Physics, Academy Str., 5 MD2028, Chisinau, Moldova; Universidad Nacional Autónoma de México, México

**Keywords:** crystal structure, azo­benzene, photoswitchers, crystal structure

## Abstract

The crystal structures of two inter­mediates, 4-amino-3,5-di­fluoro­benzo­nitrile, C_7_H_4_F_2_N_2_ (**I**), and ethyl 4-amino-3,5-di­fluoro­benzoate, C_9_H_9_F_2_NO_2_ (**II**), along with a visible-light-responsive azo­benzene derivative, diethyl 4,4′-(diazene-1,2-di­yl)bis­(3,5-di­fluoro­benzoate), C_18_H_14_F_4_N_2_O_4_ (**III**), obtained by four-step synthetic procedure, were studied using single-crystal X-ray diffraction. The mol­ecules of **I** and **II** demonstrate the quinoid character of phenyl rings accompanied by the distortion of bond angles related to the presence of fluorine substituents in the 3 and 5 (*ortho*) positions. In the crystals of **I** and **II**, the mol­ecules are connected by N—H⋯N, N—H⋯F and N—H⋯O hydrogen bonds, C—H⋯F short contacts, and π-stacking inter­actions. In crystal of **III**, only stacking inter­actions between the mol­ecules are found.

## Abstract 2

Two inter­mediates, 4-amino-3,5-di­fluoro­benzo­nitrile, C_7_H_4_F_2_N_2_ (**I**), and ethyl 4-amino-3,5-di­fluoro­benzoate, C_9_H_9_F_2_NO_2_ (**II**), along with a visible-light-responsive azo­benzene derivative, diethyl 4,4′-(diazene-1,2-di­yl)bis­(3,5-di­fluoro­benzoate), C_18_H_14_F_4_N_2_O_4_ (**III**), obtained by four-step synthetic procedure, were studied using single-crystal X-ray diffraction. In the crystals of **I** and **II**, the mol­ecules are connected by N—H⋯N, N—H⋯F and N—H⋯O hydrogen bonds, C—H⋯F short contacts, and π-stacking inter­actions. In the crystal of **III**, only stacking inter­actions between the mol­ecules are found.

## 1. Chemical context

Azo­benzene and its derivatives have different absorbance depending on the mol­ecular conformation (*trans* or *cis*) around the central N=N bond (Mostad & Rømming, 1971 ▸; Harada *et al.*, 1997 ▸) and mol­ecular architecture. Recently, derivatives of azo­benzene, known as pharmacophores, whose activity can be altered *via* the application of excitation sources with different wavelengths, started being used as mol­ecular tools for controlling biological processes (Aggarwal *et al.*, 2020 ▸; Gutzeit *et al.*, 2021 ▸). The development of new pharmacophores can be achieved *via* alteration of the mol­ecular properties by changing the chemical structure, shape, polarity, and other mol­ecular characteristics.

It was indicated (Bléger *et al.*, 2012 ▸; Knie *et al.*, 2014 ▸) that fluorination of benzene rings in azo­benzene in *ortho* positions to the N=N group along with the introduction of electron-withdrawing groups in a *para* position can help to achieve higher isomer conversion to the *cis* form when compared to other azo­benzene derivatives. In addition, it was observed that the thermal stability of the *cis* isomers of *ortho* fluorinated azo­benzenes compared to non-fluorinated materials was significantly increased from 5 h to 700 days (Bléger *et al.*, 2012 ▸).

Another inter­esting application of azo­benzene-based organic materials was presented by Peng and co-workers (Peng *et al.*, 2022 ▸), who demonstrated that the non-centrosymmetric 2-amino-2′,4,4′,6,6′-penta­fluoro­azo­benzene was a single-component ferroelectric. In that publication, it was stated that the above-mentioned material was the first single-component organic ferroelectric that opened the way to the design and exploration of azo­benzene-based ferroelectrics with promising applications in biofriendly ferroelectric devices.
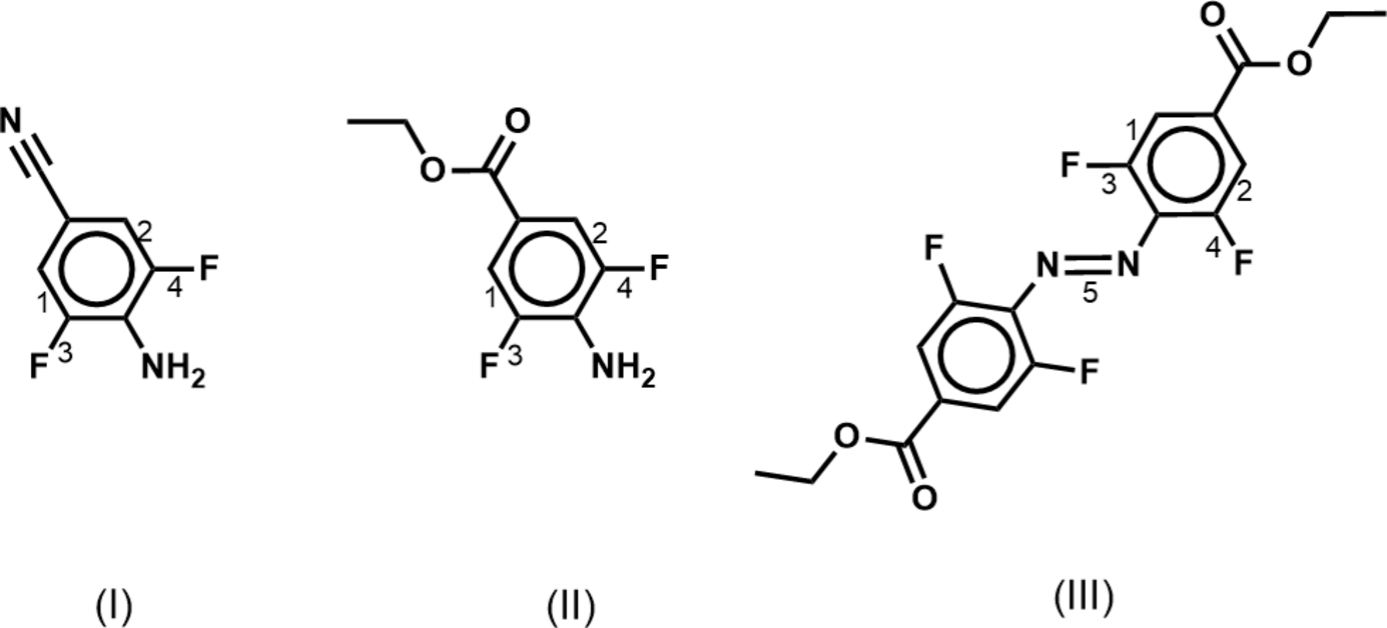


Herein, the synthetic protocols for two inter­mediates, 4-amino-3,5-di­fluoro­benzo­nitrile (**I**), ethyl 4-amino-3,5-di­fluoro­benzoate (**II**), and an azo­benzene derivative with the fluorine atoms in *ortho-*positions and ester group in a *para-* position, namely, diethyl-4,4′-(2,2′,6,6′-tetra­fluoro)­azo­benzene di­carboxyl­ate (**III**) obtained in four-step synthesis using a modified synthetic procedure (Appiah *et al.*, 2017 ▸) are reported along with the comprehensive X-ray structural study of these materials in the solid state. The synthesized azo­benzene derivative might be used as a precursor for further development and application in photopharmacological studies. It has been shown that fluorinated azo­benzenes can be used also for the synthesis of photoresponsive main-chain oligomers with azo­benzene moieties incorporated in linear unsaturated or saturated polyolefins on a gram scale (Appiah *et al.*, 2017 ▸).

## 2. Structural commentary

**Figure 1:**
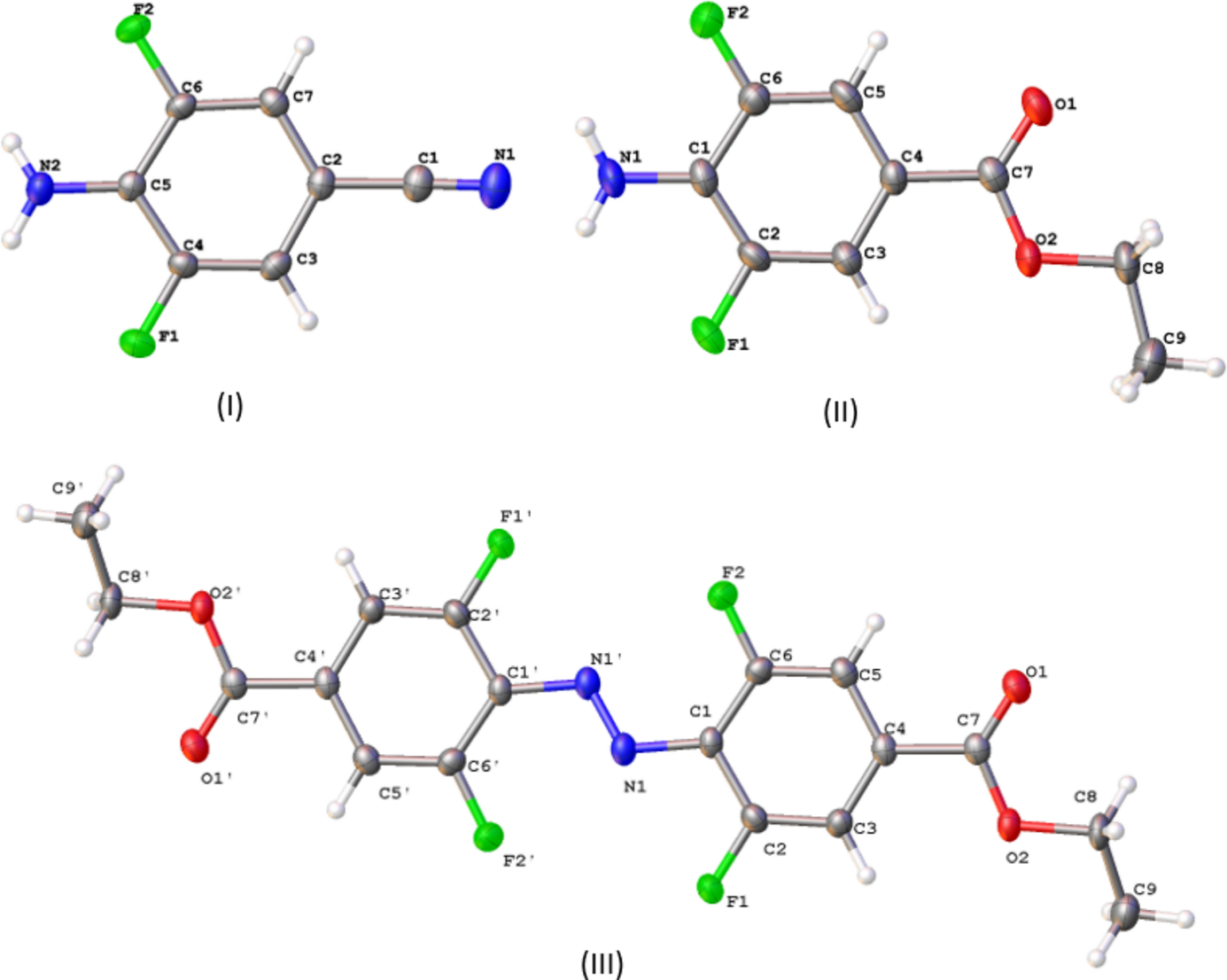
Mol­ecular structures of **I**, **II**, **III** with the atomic numbering schemes. Displacement ellipsoids are drawn at the 50% probability level. Symmetry code: (′) 1 − *x*, 1 − *y*, 2 − *z* for **III**.

The geometric parameters of mol­ecule **I** (Table 1 ▸, Fig. 1 ▸) are very similar to those found in related structures lacking fluorine substituents. For example, a comparison of the geometrical parameters of **I** with those of 4-amino­benzo­nitrile (Merlino & Sartori, 1982 ▸; Heine *et al.*, 1994 ▸; Islor *et al.*, 2013 ▸; Alimi *et al.*, 2018 ▸) in which there are no fluorine substituents, shows their similarity. However, while in 4-amino­benzo­nitrile (Alimi *et al.*, 2018 ▸), the angles in the phenyl ring are almost the same (from 118.5 to 120.8°), in **I**, the C6—C5—C4 angle with the value 114.5 (1)° is more acute than the C7—C6—C5 [124.4 (1)°] and C5—C4—C3 [124.2 (1)°] angles, mainly due to the influence of highly electronegative fluorine substituents. It also can be noted, that the cyano group bond length C1≡N1 [1.146 (2) Å] is slightly longer than the literature value (1.136 Å; Allen *et al.*, 1987 ▸). Increased conjugation can lead to a slight reduction in bond order, potentially lengthening and somewhat weakening the triple bond compared to a non-conjugated nitrile (Allen *et al.*, 1987 ▸).

Likewise, the bond lengths and angles in **II** (Table 1 ▸, Fig. 1 ▸) are very similar to those reported previously for related structures, for instance, for the mol­ecule of ethyl-4-amino­benzoate (similar to mol­ecule **II**, without fluorine substit­uents) that was reported in several publications (Lynch & McClenaghan, 2002 ▸; Chan & Welberry, 2010 ▸; Patyk-Kaźmierczak & Kaźmierczak, 2020 ▸). The mol­ecular structure in the most recent paper (Patyk-Kaźmierczak & Kaźmierczak, 2020 ▸) demonstrated equal distances of 1.390 Å between the carbon atoms in the phenyl ring.

The geometric parameters of mol­ecule **III** (Table 1 ▸, Fig. 1 ▸) are very similar to those found in related structures. There are few structures reported in the literature (Kerckhoffs *et al.*, 2022 ▸; Hermann *et al.*, 2017 ▸; Bushuyev *et al.*, 2016 ▸; Saccone *et al.*, 2014 ▸, and Aggarwal *et al.*, 2020 ▸) featuring *trans*-azo­benzene with halogen substituents at the 2- and 6-positions. However, there are two structures that have been found to incorporate the diethyl-4,4′-azo­benzene di­carboxyl­ate moiety (Niu *et al.*, 2011 ▸; Gajda *et al.*, 2014 ▸). In both those structures, the mol­ecules are planar. The title structure **III** is centrosymmetric, the phenyl rings are planar. The N1=N1′ distance [1.252 (3) Å] is very close to the distances presented in the literature. In the mol­ecule of **II**, the C7=O1 distance is 1.212 (4) Å, and in the mol­ecule of **III** the distance C7=O1 is equal to 1.211 (2) Å. Those values are close to the statistical mean value of 1.221 Å (Allen *et al.*, 1987 ▸).

For a convenient comparison of the mol­ecular geometries of **I**–**III**, some bond lengths and their notations are presented in Scheme 1 and Table 1 ▸. From Table 1 ▸, it is clear that in all mol­ecules the C—F bonds are characterized by very similar bond lengths. As a result of the *para* position of the donor and acceptor substituents of the phenyl rings, it is expected that this ring should have quinoid character (Zyss, 1994 ▸). Indeed, bond lengths 1 and 2 (see Scheme 1 and Table 1 ▸) are reduced if compared with the other bond lengths in the phenyl rings (see supporting information).

**Figure 2:**
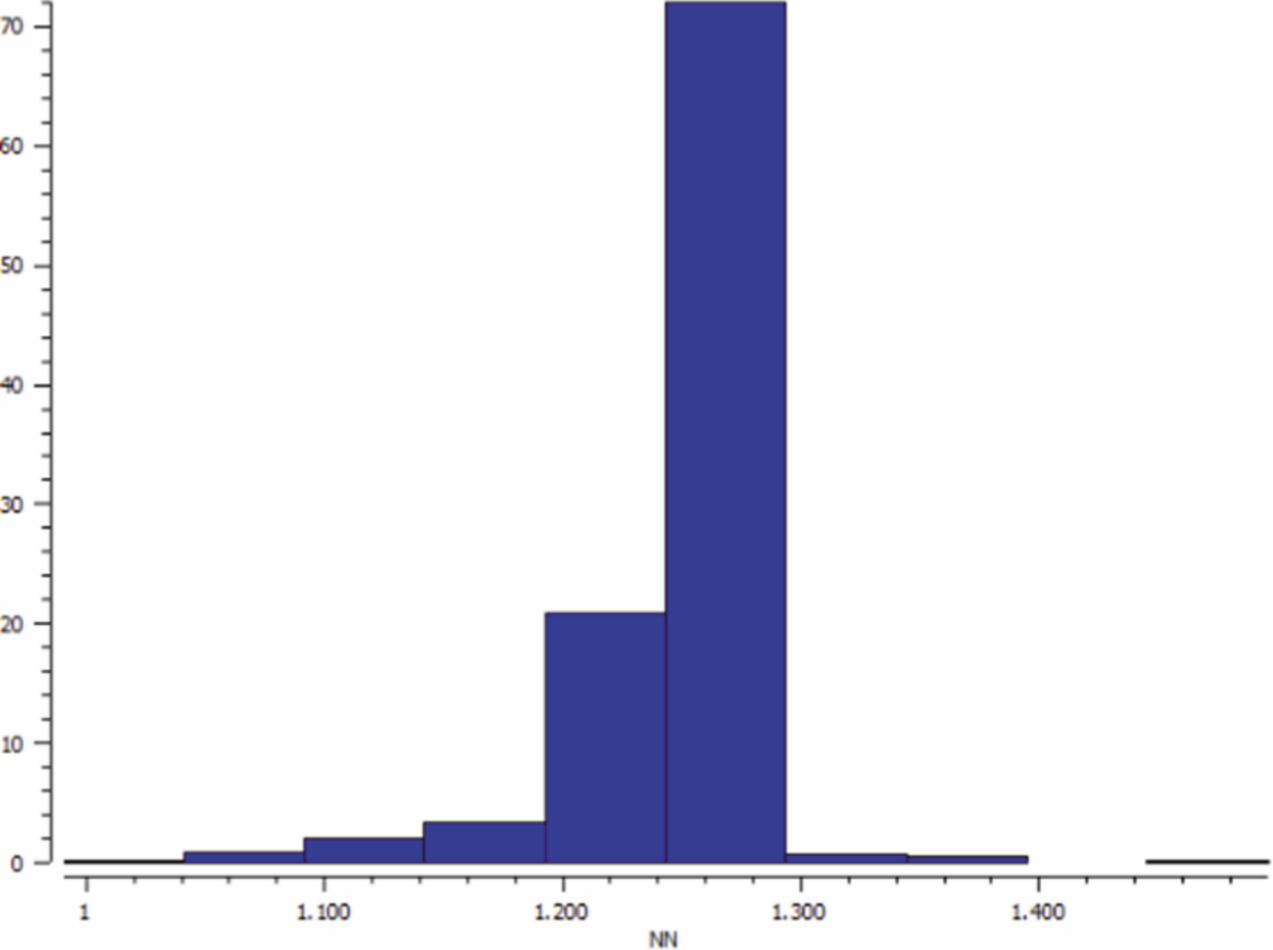
Histogram of N=N bond-length distribution in azo­benzene derivatives.

The length of the double N=N bond in mol­ecule **III** corresponds to a standard value for this series of compounds. Using CSD Version 5.45 (update of June 2024; Groom *et al.*, 2016 ▸), a statistical analysis of the N=N bonds in 2261 Ph—N=N—Ph fragments from 1733 crystal structures was carried out. A histogram of the bond-length distribution with a mean bond value of 1.246 Å, median 1.253 Å and su 0.034 Å is presented in Fig. 2 ▸. It clearly demonstrates that the central bond length in mol­ecule **III** corresponds to the median value of such bonds.

**Figure 3:**
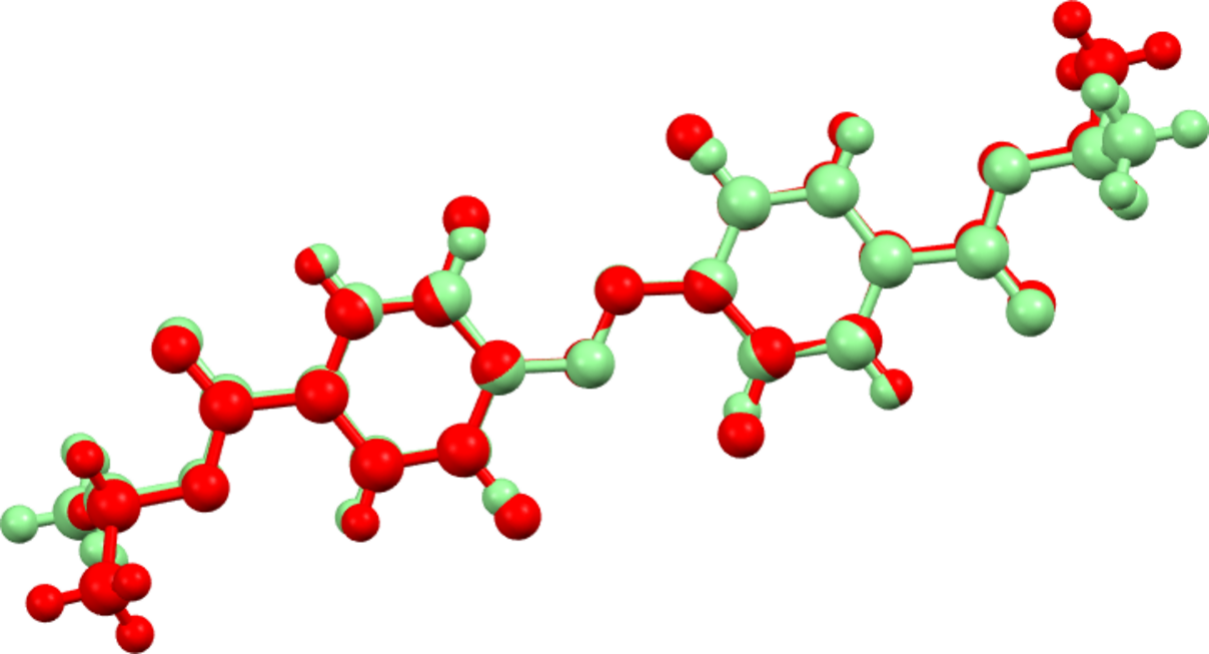
Overlay of mol­ecules **III** (red) and DDB (green) with r.m.s. 0.121.

The mol­ecule of **III**, in contrast to the previously studied mol­ecule of diethyl 4,4′-(diazenedi­yl)dibenzoate (DDB; Niu *et al.*, 2011 ▸) without F substituents, is not planar. The torsion angle that characterizes the position of the phenyl rings relative to the central C—N=N—C fragment is equal to 17.2 (3) ° in **III** and 0.2 (2) ° in DDB. It can be explained by the intra­molecular steric inter­actions between F and N atoms in **III**, N1⋯F1 = 2.644 (2) Å and N1⋯F2 = 2.945 (2) Å, which are slightly shorter than sum of van der Waals radii (Batsanov, 2001 ▸). An overlay of mol­ecules **III** and DDB demonstrates the mol­ecular similarity with the exception of the orientation of the terminal Me groups (See Fig. 3 ▸).

## 3. Supra­molecular features

**Figure 4:**
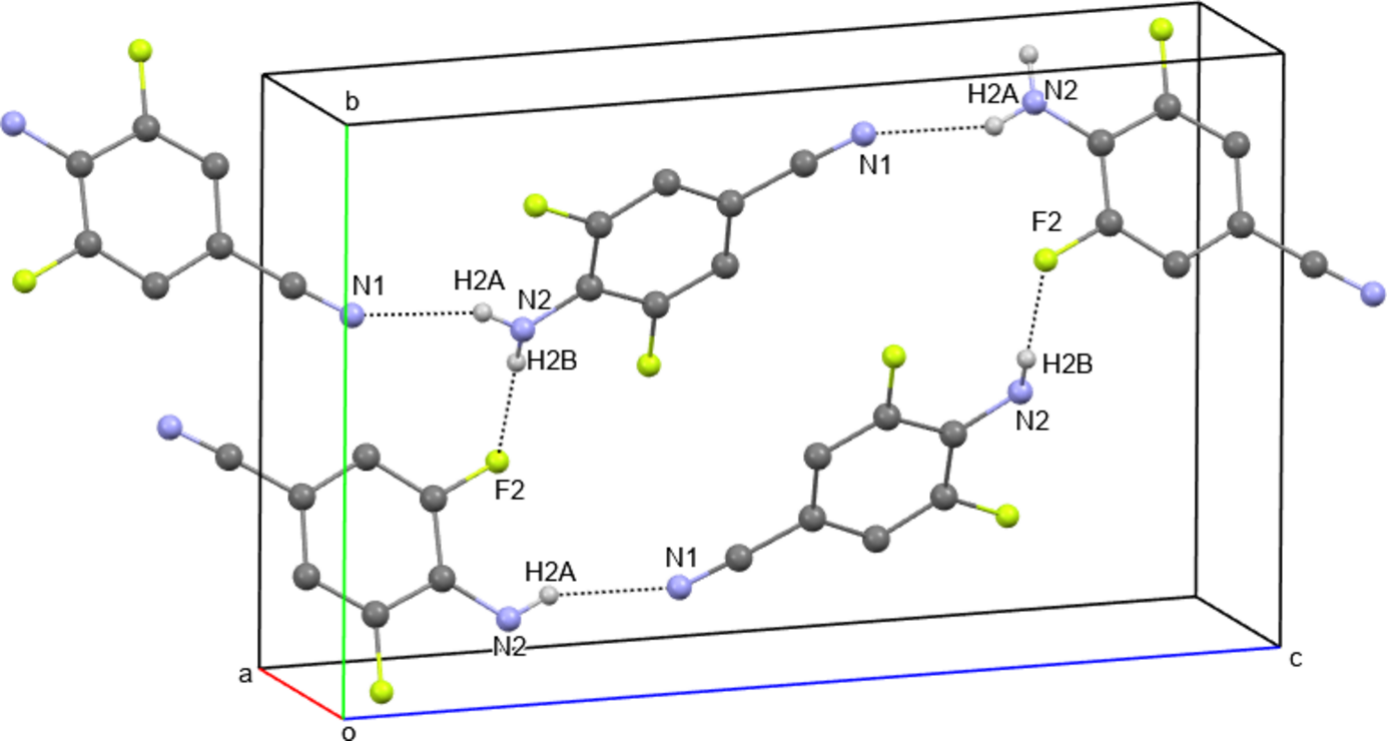
The packing in the crystal of **I**. Hydrogen bonds are shown as dotted lines. Hydrogen atoms not participating in hydrogen bonding are omitted for clarity.

The packing in the crystal of **I** is defined by weak N–H⋯N and N–H⋯F H-bonds (Fig. 4 ▸ and Table 2 ▸), and π-stacking inter­actions with inter­planar distances between the overlapping phenyl rings equal to 3.3573 (8) Å and distances between the ring centroids equal to 3.7283 (4) Å. The parameters of the short contacts correspond to the average *D*⋯*A* distances for specific inter­actions [N—H⋯N = 2.9–3.0 Å (Prasad & Govil, 1980 ▸) and N—H⋯F = 2.427 Å (Taylor, 2017 ▸)]. Inter­planar distances for stacking inter­actions are slightly shorter than the inter­planar distance in graphite (3.42 Å), indicating the significant role of stacking inter­actions in this crystal. As a result of the hydrogen bonding, mol­ecules of **I** form chains along the (101) direction.

**Figure 5:**
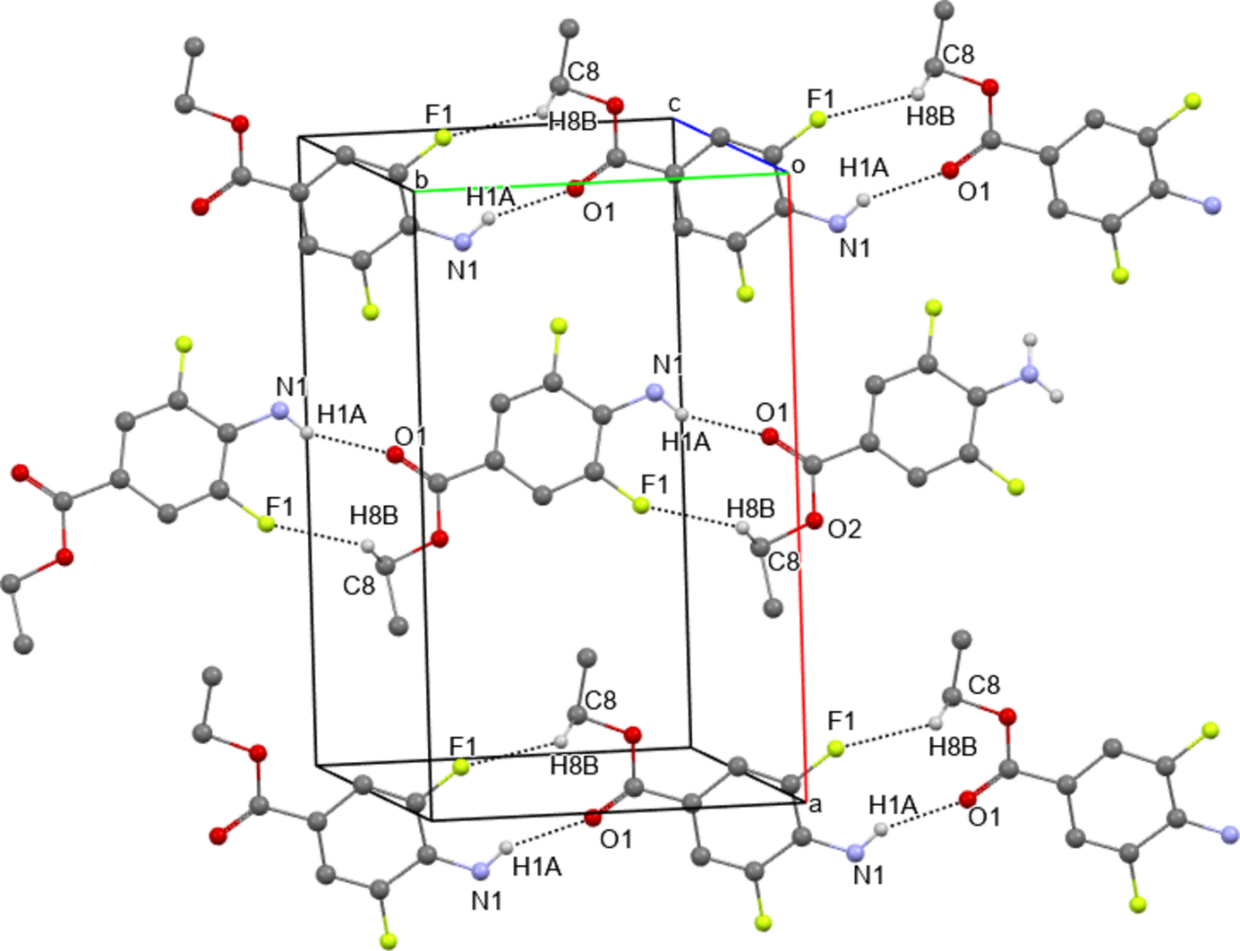
The packing in the crystal of **II**. Hydrogen bonds are shown as dashed lines. Hydrogen atoms not participating in hydrogen bonding are omitted for clarity.

In the crystal of **II**, N—H⋯O hydrogen bonds and C—H⋯F short contacts are found (Fig. 5 ▸, Table 3 ▸) as well as π–π-stacking inter­actions with inter­planar distances between phenyl rings equal to 3.325 (3) Å and distances between ring centroids of 3.490 (3) Å. The parameters of the short contacts correspond to the average *D*⋯*A* distances for specific inter­actions (N—H⋯O = 2.7–3.3 Å (Bakker *et al.*, 2023 ▸) and N—H⋯F = 2.427 (6) Å (Taylor, 2017 ▸)]. As a result of hydrogen bonding, mol­ecules of **II** form chains along the *b-*axis direction. In the structures of both **I** and **II**, short intra­molecular contacts of the type N–H⋯F are observed.

**Figure 6:**
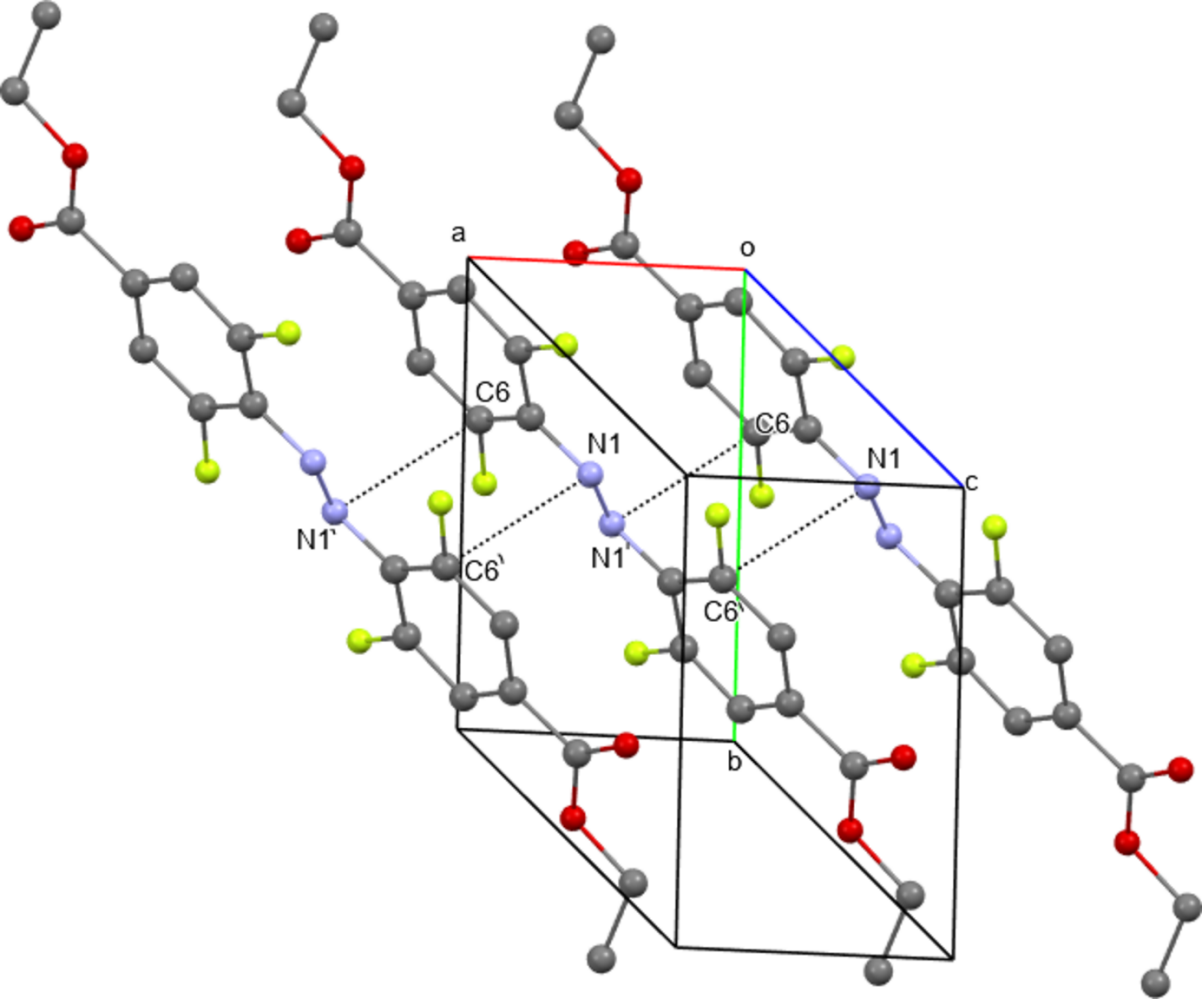
The packing in the crystal of **III**. Stacking short contacts are shown as dashed lines. Hydrogen atoms are omitted for clarity. N1⋯C6(−*x*, 1 − *y*, 2 − *z*) = 3.184 (3) Å.

The relative orientations of the mol­ecular cores in structure **III** (Fig. 6 ▸) and in the analogous crystal structure of *trans*-1,2-bis­(4-bromo-2,6-di­fluoro­phen­yl)diazene (Broichhagen *et al.*, 2015 ▸) are similar. In the crystal of **III**, the inter­planar distance between the mol­ecular core containing the phenyl rings and the N=N bond is 3.324 (13) Å and inter­centroid distance is 4.6106 (17) Å. The nearest distance of the azo group N atom to the carbon atom in the phenyl ring N1⋯C6 is 3.184 (3) Å, and the distance to the ring centroid is 3.465 (19) Å. The distance between carbonyl atom O1 and the nearest C atom in the phenyl ring is 3.316 (2) Å and to the ring centroid is 3.351 (18) Å. As a result of π–π stacking, the mol­ecules of **III** form chains along the [01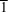] direction.

## 4. Database survey

A search of the Cambridge Structural Database (CSD version 5.45, update of June 2024; Groom *et al.*, 2016 ▸) for 4-amino-3,5-di­fluoro­benzo­nitrile (**I**) revealed that the structure had not previously been published. However, a similar structure without fluorine substituents in the ring, 4-amino-benzo­nitrile, had been described and demonstrated the possibility of different polymorphs [Alimi *et al.*, 2018 ▸ (BERTOH03); Merlino & Sartori, 1982 ▸ (BERTOH); Heine *et al.*, 1994 ▸ (BERTOH01); Islor *et al.*, 2013 ▸ (BERTOH02)] all of which crystallize in centrosymmetric space groups, except for BERTOH01 (Heine *et al.*, 1994 ▸) which crystallizes in the non-centrosymmetric *P*2_1_2_1_2_1_ space group.

The novelty of 4-amino-3,5-di­fluoro­benzoate (**II**) was also confirmed by the lack of this structure in the CSD. The closest analogue without fluorine substituents in the phenyl ring is ethyl 4-amino-benzoate (benzocaine), an anesthetic applied in medicine and the pharmaceutical industry and described as 19 database entries. Eight of the structures were reported by Patyk-Kaźmierczak & Kaźmierczak (2020 ▸) (QQQAXG11–18), which represent several polymorphs in the same *P*2_1_/*c* space group but with different cell parameters. Other structures were reported by Patel *et al.* (2017 ▸) (QQQAXG09–10) where two polymorphs of benzocaine were described in space groups *P*2_1_2_1_2_1_ and *P*2_1_/*c*. Earlier, these structures were mentioned by Chan & Welberry (2010 ▸) and Lynch & McClenaghan (2002 ▸).

The structure of diethyl-4,4′-(2,2′,6,6′-tetra­fluoro)­azo­benzene di­carboxyl­ate (**III**) had also not been deposited in the CSD. However, similar *tetra-*halogenated mol­ecules with the halogens in *ortho* positions have been described (Kerckhoffs *et al.*, 2022 ▸; TETROD). It should be mentioned that mol­ecules of *cis* and *trans* (*Z* and *E*) isomers were structurally characterized when it was possible to separate them and when the *cis* isomers had a long half-life to facilitate their isolation. The authors showed that it was possible to modify the stability of the *cis* isomers by introducing larger halogen atoms in the *ortho* positions and a heavier element substituent in the *para* position. If the azo-benzene mol­ecules with small substituents (H, F) in the *ortho* positions are planar, with larger halogens (I) they are non-planar, and their *cis* isomers are more stable. It is important to mention that the *ortho*-tetra­fluoro­azo­benzenes (DIQBOX; Hermann *et al.*, 2017 ▸) resulting from isomerization of the mol­ecule, resulted in significant shape changes, with the *trans* isomer exhibiting elongation and the *cis* isomer adopting a more spherical shape. In addition, diethyl-4,4′-azo­benzene di­carboxyl­ate, which represents the non-halogenated analogue of compound **III** [Niu *et al.*, 2011 ▸ (AZUKAI); Gajda *et al.*, 2014 ▸ (AZUKAI01)], has a planar arrangement in the *trans* isomers. However, if the *ortho* positions are occupied by bulky iodine substituents, such as in diethyl 4,4′-diazenediylbis(3,5-di­iodo­benzoate), the mol­ecule is non-planar in the *trans* form (TETROD; Kerckhoffs *et al.*, 2022 ▸). In addition, some non-halogenated and halogenated azo­benzenes, both *cis* and *trans* isomers, have been studied [Hampson & Robertson,1941 ▸ (AZBENC); Mostad *et al.*, 1971 ▸ (AZBENC01); De Lange *et al.*, 1939 ▸ (AZOBEN); Chinnakali *et al.*, 1993 ▸ (WACHAJ); Harada *et al.*, 1997 ▸ (AZOBEN04–06; Bushuyev *et al.*, 2016 ▸ (SUWKIG); Saccone *et al.*, 2014 ▸ (PINLUV); Aggarwal *et al.*, 2020 ▸ (TUXNEI and TUXNAE)].

## 5. Synthesis and crystallization

The synthesis of mol­ecules **I**–**III** is shown in the reaction scheme, and follows a slight modification of the procedure described previously (Appiah *et al.*, 2017 ▸). Starting materials were purchased from Ambeed Inc. and Sigma-Aldrich and used without further purification. To obtain 4-amino-3,5-di­fluoro­benzo­nitrile (**I**), 4-bromo-2,6-di­fluoro­aniline (50.0 g, 240 mmol, 1 eq.) and CuCN (64.5 g, 720 mmol, 3 eq.) were suspended in di­methyl­formamide (DMF, 500 mL) and refluxed for 24 h. The mixture was cooled to room temperature and NH_4_OH (2 L, 18%) was added, and the resulting solution was filtered. The mixture (filtrate) was extracted with EtOAc (4 × 750 mL) and the organic phase was washed with NH_4_OH 18%, de-ionized water, brine, dried with Na_2_SO_4_, and filtered. The residue was purified through a silica gel plug with CH_2_Cl_2_/*n*-hexane 2:1, to yield a dark-brown solid (15.7 g, 102 mmol, 42% yield).
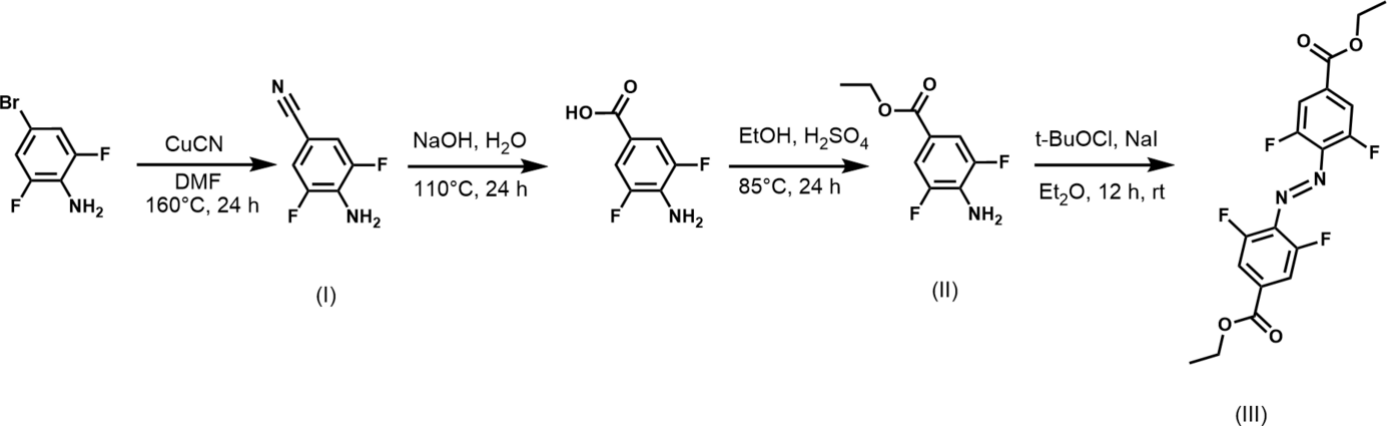


The synthesis of 4-amino-3,5-di­fluoro­benzoic acid was conducted by treating 4-amino-3,5-di­fluoro­benzo­nitrile (14.91 g, 96.7 mmol, 1 eq.) with sodium hydroxide (NaOH 1 *M*, 480 mL). The resulting solution was heated to reflux for 24 h. The reaction was then cooled to room temperature and HCl conc. (60 mL) was added to the reaction mixture dropwise until the reaction turned acidic pH ∼1; the product precipitated as a hydro­chloride salt. The salt was then dissolved in ethyl acetate, dried over MgSO_4_, filtered, and concentrated under vacuum to obtain 4-amino-3,5-di­fluoro­benzoic acid (14.9 g, 81.4 mmol, 84.2%).

To obtain 4-amino-3,5-di­fluoro­benzoate (**II**), 4-amino-3,5-di­fluoro­benzoic acid (14.9 g, 96.7 mmol) was dissolved in ethanol (300 mL) and H_2_SO_4_ (6 mL) and refluxed for 10 h. The reaction was neutralized using a saturated solution of sodium bicarbonate, followed by extraction with di­chloro­methane (DCM, 4 × 300 mL). The organic phase was dried using sodium sulfate (Na_2_SO_4_), filtered, and concentrated under reduced pressure, yielding the inter­mediate product (14.99 g, 75 mmol, 77% yield).

To obtain diethyl-4,4′-(2,2′,6,6′-tetra­fluoro)­azo­benzene di­carboxyl­ate (**III**), 4-amino-3,5-di­fluoro­benzoate (12.0 g, 60 mmol, 1 eq.) and sodium iodide (NaI) (18.4 g, 120 mmol, 2 eq.) in Et_2_O (400 mL) were added into a 1 L flask. To the reaction mixture, *tert*-butyl hypochlorite (*t*-BuOCl, 14 mL, 4 eq.) was added and the resulting mixture was stirred for 12 h at rt. Thereafter, a freshly prepared solution of 1 *M* Na_2_SO_3_ (1200 mL) was added, and the mixture was mixed thoroughly. The resulting mixture was washed by DCM (1 × 600 mL) and the organic layer was collected and washed with RO water (3 × 1L) and brine (3 x 750 mL), dried with Na_2_SO_4_ (anhydrous), filtered, purified through a silica gel plug and evaporated under reduced pressure. The crude product was rinsed with a small amount of EtOAc to yield a reddish precipitate (4.9 g, 12.4 mmol, 21% yield).

Crystallization of all compounds for diffraction studies was performed using the slow evaporation method. All solutions were prepared by dissolving compounds **I**–**III** in DCM (2 mL) and sonicating them for 10 min. Then they were capped with cotton plugs and left in the hood for 4 days. Thereafter transparent plate-like crystals of **I** and **II**, and dark-red needle-like crystals of **III** were obtained.

## 6. Refinement

Crystal data, data collection and structure refinement details for compounds **I, II**, and **III** are summarized in Table 4 ▸. The acidic N–H protons in **I** and **II** were localized from the residual electron-density map and refined freely. All other H atoms were positioned geometrically (C—H = 0.95–0.99 Å) and refined as riding with *U*_iso_(H) = 1.2*U*_eq_(C) or 1.5*U*_eq_(C-meth­yl).

## Supplementary Material

Crystal structure: contains datablock(s) I, II, III. DOI: 10.1107/S2056989024006819/jq2034sup1.cif

Structure factors: contains datablock(s) I. DOI: 10.1107/S2056989024006819/jq2034Isup4.hkl

Structure factors: contains datablock(s) II. DOI: 10.1107/S2056989024006819/jq2034IIsup3.hkl

Structure factors: contains datablock(s) III. DOI: 10.1107/S2056989024006819/jq2034IIIsup2.hkl

Supporting information file. DOI: 10.1107/S2056989024006819/jq2034Isup5.cml

Supporting information file. DOI: 10.1107/S2056989024006819/jq2034IIsup6.cml

Supporting information file. DOI: 10.1107/S2056989024006819/jq2034IIIsup7.cml

CCDC references: 2370064, 2370063, 2370062

Additional supporting information:  crystallographic information; 3D view; checkCIF report

## Figures and Tables

**Table 1:** Selected bond lengths (Å) in mol­ecules of **I**–**III**

Bond/Compound	**I**	**II**	**III**
1	1.373 (2)	1.372 (4)	1.373 (3)
2	1.377 (2)	1.365 (4)	1.373 (3)
3	1.360 (1)	1.360 (3)	1.345 (2)
4	1.360 (1)	1.368 (3)	1.341 (2)
5	–	–	1.252 (3)

**Table 2:** Hydrogen-bond geometry (Å, °) for **I**[Chem scheme1]

*D*—H⋯*A*	*D*—H	H⋯*A*	*D*⋯*A*	*D*—H⋯*A*
N2—H2*A*⋯N1^i^	0.82 (2)	2.28 (2)	3.0297 (17)	153 (2)
N2—H2*B*⋯F2^ii^	0.88 (2)	2.38 (2)	3.2526 (15)	173.9 (19)

Symmetry codes: (i) 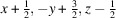; (ii) 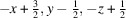.

**Table 3:** Hydrogen-bond geometry (Å, °) for **II**[Chem scheme1]

*D*—H⋯*A*	*D*—H	H⋯*A*	*D*⋯*A*	*D*—H⋯*A*
N1—H1*A*⋯O1^i^	0.85 (3)	2.15 (4)	2.942 (3)	155 (3)
C8—H8*B*⋯F1^ii^	0.99	2.46	3.015 (3)	115

Symmetry codes: (i) 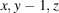; (ii) 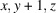.

**Table 4:** Experimental details

	**I**	**II**	**III**
Crystal data			
Chemical formula	C_7_H_4_F_2_N_2_	C_9_H_9_F_2_NO_2_	C_18_H_14_F_4_N_2_O_4_
*M*_r_	154.12	201.17	398.31
Crystal system, space group	Monoclinic, *P*2_1_/*n*	Orthorhombic, *Pbcn*	Triclinic, *P* 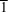
Temperature (K)	100	100	100
*a*, *b*, *c* (Å)	3.7283 (4), 10.5275 (12), 16.9073 (19)	14.877 (3), 8.9995 (18), 13.635 (3)	4.6106 (17), 8.839 (3), 10.969 (4)
α, β, γ (°)	90, 94.604 (2), 90	90, 90, 90	99.330 (8), 99.431 (8), 96.442 (7)
*V* (Å^3^)	661.46 (13)	1825.6 (6)	430.7 (3)
*Z*	4	8	1
Radiation type	Mo *K*α	Mo *K*α	Mo *K*α
μ (mm^−1^)	0.14	0.13	0.14
Crystal size (mm)	0.36 × 0.22 × 0.12	0.21 × 0.13 × 0.11	0.57 × 0.13 × 0.1

Data collection			
Diffractometer	Bruker APEXII CCD	Bruker APEXII CCD	Bruker *SMART* APEXII
Absorption correction	Multi-scan (*SADABS*; Krause *et al.*, 2015 ▸)	Multi-scan (*SADABS*; Krause *et al.*, 2015 ▸)	Multi-scan (*SADABS*; Krause *et al.*, 2015 ▸)
*T*_min_, *T*_max_	0.661, 0.746	0.973, 0.986	0.926, 0.986
No. of measured, independent and observed [*I* > 2σ(*I*)] reflections	11242, 2140, 1775	29363, 1665, 1265	3269, 1913, 1414
*R*_int_	0.029	0.114	0.023
(sin θ/λ)_max_ (Å^−1^)	0.738	0.600	0.649

Refinement			
*R*[*F*^2^ > 2σ(*F*^2^)], *wR*(*F*^2^), *S*	0.047, 0.130, 1.09	0.060, 0.131, 1.21	0.047, 0.150, 1.01
No. of reflections	2140	1665	1913
No. of parameters	108	136	132
H-atom treatment	H atoms treated by a mixture of independent and constrained refinement	H atoms treated by a mixture of independent and constrained refinement	H atoms treated by a mixture of independent and constrained refinement
Δρ_max_, Δρ_min_ (e Å^−3^)	0.46, −0.23	0.20, −0.27	0.28, −0.29

Computer programs: *APEX2* and *SAINT-Plus* (Bruker, 2019 ▸), SHELXT (Sheldrick, 2015*a* ▸), *SHELXS* (Sheldrick, 2008 ▸), *SHELXL* (Sheldrick, 2015*b* ▸) and *OLEX2* (Dolomanov *et al.*, 2009 ▸).

## supplementary crystallographic information

### 4-Amino-3,5-difluorobenzonitrile (I) Crystal data

**Table:**

C_7_H_4_F_2_N_2_	*F*(000) = 312
*M**_r_* = 154.12	*D*_x_ = 1.548 Mg m^−^^3^
Monoclinic, *P*2_1_/*n*	Mo *K*α radiation, λ = 0.71073 Å
*a* = 3.7283 (4) Å	Cell parameters from 4913 reflections
*b* = 10.5275 (12) Å	θ = 2.3–31.2°
*c* = 16.9073 (19) Å	µ = 0.14 mm^−^^1^
β = 94.604 (2)°	*T* = 100 K
*V* = 661.46 (13) Å^3^	Block, clear colourless
*Z* = 4	0.36 × 0.22 × 0.12 mm

### 4-Amino-3,5-difluorobenzonitrile (I) Data collection

**Table:**

Bruker APEXII CCD diffractometer	2140 independent reflections
Radiation source: sealed X-ray tube, EIGENMANN GmbH	1775 reflections with *I* > 2σ(*I*)
Graphite monochromator	*R*_int_ = 0.029
Detector resolution: 7.9 pixels mm^-1^	θ_max_ = 31.7°, θ_min_ = 2.3°
φ and ω scans	*h* = −5→5
Absorption correction: multi-scan (SADABS; Krause *et al.*, 2015)	*k* = −14→15
*T*_min_ = 0.661, *T*_max_ = 0.746	*l* = −23→24
11242 measured reflections	

### 4-Amino-3,5-difluorobenzonitrile (I) Refinement

**Table:**

Refinement on *F*^2^	Primary atom site location: dual
Least-squares matrix: full	Hydrogen site location: mixed
*R*[*F*^2^ > 2σ(*F*^2^)] = 0.047	H atoms treated by a mixture of independent and constrained refinement
*wR*(*F*^2^) = 0.130	*w* = 1/[σ^2^(*F*_o_^2^) + (0.0659*P*)^2^ + 0.2291*P*] where *P* = (*F*_o_^2^ + 2*F*_c_^2^)/3
*S* = 1.09	(Δ/σ)_max_ < 0.001
2140 reflections	Δρ_max_ = 0.46 e Å^−^^3^
108 parameters	Δρ_min_ = −0.23 e Å^−^^3^
0 restraints	

### 4-Amino-3,5-difluorobenzonitrile (I) Special details

**Table:**

Geometry. All esds (except the esd in the dihedral angle between two l.s. planes) are estimated using the full covariance matrix. The cell esds are taken into account individually in the estimation of esds in distances, angles and torsion angles; correlations between esds in cell parameters are only used when they are defined by crystal symmetry. An approximate (isotropic) treatment of cell esds is used for estimating esds involving l.s. planes.

### 4-Amino-3,5-difluorobenzonitrile (I) Fractional atomic coordinates and isotropic or equivalent isotropic displacement parameters (Å^2^)

**Table:**

	*x*	*y*	*z*	*U*_iso_*/*U*_eq_	
F1	0.8337 (2)	0.47654 (7)	0.39927 (5)	0.0311 (2)	
F2	0.3651 (2)	0.80481 (8)	0.23493 (4)	0.0314 (2)	
N2	0.6762 (3)	0.56770 (11)	0.24999 (7)	0.0276 (3)	
N1	0.2518 (4)	0.89360 (14)	0.57454 (7)	0.0379 (3)	
C6	0.4379 (3)	0.75726 (11)	0.30925 (7)	0.0206 (2)	
C4	0.6741 (3)	0.59252 (11)	0.39189 (7)	0.0207 (2)	
C5	0.6003 (3)	0.63732 (11)	0.31445 (6)	0.0194 (2)	
C2	0.4317 (3)	0.77685 (12)	0.44923 (7)	0.0217 (2)	
C7	0.3518 (3)	0.82791 (11)	0.37357 (7)	0.0215 (2)	
H7	0.241446	0.908931	0.366727	0.026*	
C3	0.5960 (3)	0.65780 (12)	0.45866 (7)	0.0224 (2)	
H3	0.652163	0.622977	0.509999	0.027*	
C1	0.3337 (4)	0.84385 (13)	0.51849 (8)	0.0267 (3)	
H2A	0.671 (6)	0.602 (2)	0.2066 (14)	0.047 (6)*	
H2B	0.814 (5)	0.500 (2)	0.2560 (12)	0.036 (5)*	

### 4-Amino-3,5-difluorobenzonitrile (I) Atomic displacement parameters (Å^2^)

**Table:**

	*U*^11^	*U*^22^	*U*^33^	*U*^12^	*U*^13^	*U*^23^
F1	0.0404 (5)	0.0221 (4)	0.0309 (4)	0.0075 (3)	0.0035 (3)	0.0050 (3)
F2	0.0466 (5)	0.0272 (4)	0.0199 (4)	0.0021 (3)	−0.0008 (3)	0.0057 (3)
N2	0.0384 (6)	0.0244 (5)	0.0208 (5)	0.0015 (5)	0.0065 (4)	−0.0020 (4)
N1	0.0425 (7)	0.0426 (7)	0.0299 (6)	−0.0056 (6)	0.0099 (5)	−0.0095 (5)
C6	0.0227 (5)	0.0204 (5)	0.0185 (5)	−0.0036 (4)	0.0008 (4)	0.0032 (4)
C4	0.0214 (5)	0.0173 (5)	0.0236 (5)	−0.0008 (4)	0.0025 (4)	0.0022 (4)
C5	0.0190 (5)	0.0192 (5)	0.0202 (5)	−0.0045 (4)	0.0034 (4)	−0.0001 (4)
C2	0.0201 (5)	0.0240 (6)	0.0213 (5)	−0.0049 (4)	0.0040 (4)	−0.0031 (4)
C7	0.0208 (5)	0.0185 (5)	0.0252 (5)	−0.0011 (4)	0.0023 (4)	−0.0008 (4)
C3	0.0231 (5)	0.0256 (6)	0.0187 (5)	−0.0037 (4)	0.0017 (4)	0.0029 (4)
C1	0.0267 (6)	0.0286 (6)	0.0253 (6)	−0.0056 (5)	0.0046 (4)	−0.0034 (5)

### 4-Amino-3,5-difluorobenzonitrile (I) Geometric parameters (Å, º)

**Table:**

F1—C4	1.3596 (14)	C4—C5	1.3982 (16)
F2—C6	1.3596 (13)	C4—C3	1.3726 (16)
N2—C5	1.3617 (15)	C2—C7	1.3975 (17)
N2—H2A	0.82 (2)	C2—C3	1.3984 (17)
N2—H2B	0.88 (2)	C2—C1	1.4390 (17)
N1—C1	1.1455 (18)	C7—H7	0.9500
C6—C5	1.4000 (16)	C3—H3	0.9500
C6—C7	1.3765 (16)		
			
C5—N2—H2A	119.3 (15)	C4—C5—C6	114.49 (10)
C5—N2—H2B	120.1 (13)	C7—C2—C3	120.55 (11)
H2A—N2—H2B	115.7 (19)	C7—C2—C1	120.46 (11)
F2—C6—C5	116.31 (10)	C3—C2—C1	118.95 (11)
F2—C6—C7	119.29 (11)	C6—C7—C2	117.98 (11)
C7—C6—C5	124.39 (11)	C6—C7—H7	121.0
F1—C4—C5	116.14 (10)	C2—C7—H7	121.0
F1—C4—C3	119.63 (10)	C4—C3—C2	118.35 (10)
C3—C4—C5	124.23 (11)	C4—C3—H3	120.8
N2—C5—C6	123.49 (11)	C2—C3—H3	120.8
N2—C5—C4	122.01 (11)	N1—C1—C2	177.81 (15)
			
F1—C4—C5—N2	1.45 (17)	C7—C6—C5—N2	178.51 (12)
F1—C4—C5—C6	−179.65 (10)	C7—C6—C5—C4	−0.36 (17)
F1—C4—C3—C2	−179.98 (10)	C7—C2—C3—C4	−0.41 (17)
F2—C6—C5—N2	−1.79 (17)	C3—C4—C5—N2	−178.59 (12)
F2—C6—C5—C4	179.34 (10)	C3—C4—C5—C6	0.31 (17)
F2—C6—C7—C2	−179.65 (10)	C3—C2—C7—C6	0.36 (17)
C5—C6—C7—C2	0.04 (18)	C1—C2—C7—C6	−177.28 (11)
C5—C4—C3—C2	0.06 (18)	C1—C2—C3—C4	177.26 (11)

### 4-Amino-3,5-difluorobenzonitrile (I) Hydrogen-bond geometry (Å, º)

**Table:**

*D*—H···*A*	*D*—H	H···*A*	*D*···*A*	*D*—H···*A*
N2—H2*A*···N1^i^	0.82 (2)	2.28 (2)	3.0297 (17)	153 (2)
N2—H2*B*···F2^ii^	0.88 (2)	2.38 (2)	3.2526 (15)	173.9 (19)

Symmetry codes: (i) *x*+1/2, −*y*+3/2, *z*−1/2; (ii) −*x*+3/2, *y*−1/2, −*z*+1/2.

### Ethyl 4-amino-3,5-difluorobenzoate (II) Crystal data

**Table:**

C_9_H_9_F_2_NO_2_	*D*_x_ = 1.464 Mg m^−^^3^
*M**_r_* = 201.17	Mo *K*α radiation, λ = 0.71073 Å
Orthorhombic, *P**b**c**n*	Cell parameters from 3255 reflections
*a* = 14.877 (3) Å	θ = 2.7–23.4°
*b* = 8.9995 (18) Å	µ = 0.13 mm^−^^1^
*c* = 13.635 (3) Å	*T* = 100 K
*V* = 1825.6 (6) Å^3^	Block, clear colourless
*Z* = 8	0.21 × 0.13 × 0.11 mm
*F*(000) = 832	

### Ethyl 4-amino-3,5-difluorobenzoate (II) Data collection

**Table:**

Bruker APEXII CCD diffractometer	1665 independent reflections
Radiation source: sealed X-ray tube, EIGENMANN GmbH	1265 reflections with *I* > 2σ(*I*)
Graphite monochromator	*R*_int_ = 0.114
Detector resolution: 7.9 pixels mm^-1^	θ_max_ = 25.3°, θ_min_ = 2.7°
ω and φ scans	*h* = −17→17
Absorption correction: multi-scan (SADABS; Krause *et al.*, 2015)	*k* = −10→10
*T*_min_ = 0.973, *T*_max_ = 0.986	*l* = −16→16
29363 measured reflections	

### Ethyl 4-amino-3,5-difluorobenzoate (II) Refinement

**Table:**

Refinement on *F*^2^	0 restraints
Least-squares matrix: full	Hydrogen site location: mixed
*R*[*F*^2^ > 2σ(*F*^2^)] = 0.060	H atoms treated by a mixture of independent and constrained refinement
*wR*(*F*^2^) = 0.131	*w* = 1/[σ^2^(*F*_o_^2^) + 3.0535*P*] where *P* = (*F*_o_^2^ + 2*F*_c_^2^)/3
*S* = 1.21	(Δ/σ)_max_ < 0.001
1665 reflections	Δρ_max_ = 0.20 e Å^−^^3^
136 parameters	Δρ_min_ = −0.26 e Å^−^^3^

### Ethyl 4-amino-3,5-difluorobenzoate (II) Special details

**Table:**

Geometry. All esds (except the esd in the dihedral angle between two l.s. planes) are estimated using the full covariance matrix. The cell esds are taken into account individually in the estimation of esds in distances, angles and torsion angles; correlations between esds in cell parameters are only used when they are defined by crystal symmetry. An approximate (isotropic) treatment of cell esds is used for estimating esds involving l.s. planes.

### Ethyl 4-amino-3,5-difluorobenzoate (II) Fractional atomic coordinates and isotropic or equivalent isotropic displacement parameters (Å^2^)

**Table:**

	*x*	*y*	*z*	*U*_iso_*/*U*_eq_	
F1	0.54938 (12)	0.31169 (18)	0.35054 (15)	0.0396 (5)	
F2	0.26856 (12)	0.4861 (2)	0.45376 (15)	0.0403 (5)	
O2	0.58679 (14)	0.8516 (2)	0.34897 (17)	0.0327 (6)	
O1	0.45423 (15)	0.9500 (2)	0.39457 (17)	0.0365 (6)	
N1	0.3767 (2)	0.2502 (3)	0.4064 (2)	0.0374 (8)	
C6	0.35426 (19)	0.5153 (3)	0.4228 (2)	0.0284 (7)	
C1	0.4077 (2)	0.3925 (3)	0.4014 (2)	0.0275 (7)	
C4	0.4707 (2)	0.6876 (3)	0.3866 (2)	0.0253 (7)	
C2	0.4942 (2)	0.4276 (3)	0.3721 (2)	0.0274 (7)	
C7	0.5012 (2)	0.8429 (3)	0.3781 (2)	0.0278 (7)	
C3	0.5275 (2)	0.5693 (3)	0.3645 (2)	0.0279 (7)	
H3	0.587797	0.586326	0.344706	0.033*	
C5	0.3829 (2)	0.6589 (3)	0.4160 (2)	0.0279 (7)	
H5	0.343351	0.738484	0.431279	0.034*	
C8	0.6241 (2)	1.0007 (3)	0.3392 (3)	0.0350 (8)	
H8A	0.625203	1.051356	0.403614	0.042*	
H8B	0.587471	1.060564	0.293261	0.042*	
C9	0.7174 (2)	0.9825 (4)	0.3006 (3)	0.0468 (10)	
H9A	0.715460	0.928930	0.238005	0.070*	
H9B	0.753448	0.926045	0.347797	0.070*	
H9C	0.744541	1.080539	0.290498	0.070*	
H1A	0.414 (2)	0.179 (4)	0.399 (3)	0.043 (11)*	
H1B	0.326 (3)	0.237 (4)	0.435 (3)	0.045 (12)*	

### Ethyl 4-amino-3,5-difluorobenzoate (II) Atomic displacement parameters (Å^2^)

**Table:**

	*U*^11^	*U*^22^	*U*^33^	*U*^12^	*U*^13^	*U*^23^
F1	0.0434 (11)	0.0199 (9)	0.0556 (13)	0.0081 (8)	0.0050 (10)	−0.0026 (9)
F2	0.0312 (10)	0.0295 (10)	0.0603 (13)	−0.0029 (8)	0.0050 (9)	0.0013 (9)
O2	0.0363 (13)	0.0176 (10)	0.0442 (14)	−0.0032 (9)	0.0032 (10)	0.0027 (10)
O1	0.0390 (13)	0.0195 (11)	0.0512 (16)	0.0044 (10)	−0.0015 (11)	−0.0011 (10)
N1	0.0405 (19)	0.0169 (14)	0.055 (2)	−0.0022 (14)	0.0048 (16)	0.0004 (13)
C6	0.0250 (16)	0.0267 (17)	0.0335 (18)	−0.0001 (13)	−0.0012 (14)	0.0017 (14)
C1	0.0352 (18)	0.0190 (15)	0.0284 (18)	−0.0012 (13)	−0.0053 (14)	0.0001 (13)
C4	0.0285 (17)	0.0198 (15)	0.0276 (17)	−0.0003 (12)	−0.0048 (13)	0.0021 (12)
C2	0.0293 (17)	0.0194 (15)	0.0336 (18)	0.0084 (13)	−0.0008 (14)	−0.0018 (13)
C7	0.0329 (18)	0.0240 (16)	0.0264 (17)	0.0015 (14)	−0.0049 (14)	−0.0017 (14)
C3	0.0302 (18)	0.0236 (16)	0.0299 (18)	0.0021 (13)	0.0002 (13)	−0.0011 (13)
C5	0.0326 (18)	0.0191 (15)	0.0321 (18)	0.0082 (13)	−0.0054 (14)	−0.0014 (13)
C8	0.044 (2)	0.0182 (15)	0.043 (2)	−0.0047 (15)	−0.0021 (16)	0.0007 (14)
C9	0.045 (2)	0.035 (2)	0.060 (3)	−0.0108 (17)	0.0055 (19)	0.0014 (18)

### Ethyl 4-amino-3,5-difluorobenzoate (II) Geometric parameters (Å, º)

**Table:**

F1—C2	1.360 (3)	C4—C3	1.392 (4)
F2—C6	1.368 (3)	C4—C5	1.390 (4)
O2—C7	1.336 (4)	C2—C3	1.372 (4)
O2—C8	1.458 (4)	C3—H3	0.9500
O1—C7	1.212 (4)	C5—H5	0.9500
N1—C1	1.363 (4)	C8—H8A	0.9900
N1—H1A	0.86 (4)	C8—H8B	0.9900
N1—H1B	0.86 (4)	C8—C9	1.493 (5)
C6—C1	1.392 (4)	C9—H9A	0.9800
C6—C5	1.365 (4)	C9—H9B	0.9800
C1—C2	1.385 (4)	C9—H9C	0.9800
C4—C7	1.474 (4)		
			
C7—O2—C8	116.4 (2)	C4—C3—H3	120.8
C1—N1—H1A	118 (2)	C2—C3—C4	118.4 (3)
C1—N1—H1B	117 (2)	C2—C3—H3	120.8
H1A—N1—H1B	122 (3)	C6—C5—C4	119.3 (3)
F2—C6—C1	116.4 (3)	C6—C5—H5	120.4
C5—C6—F2	119.6 (3)	C4—C5—H5	120.4
C5—C6—C1	124.0 (3)	O2—C8—H8A	110.4
N1—C1—C6	122.9 (3)	O2—C8—H8B	110.4
N1—C1—C2	122.9 (3)	O2—C8—C9	106.6 (3)
C2—C1—C6	114.2 (3)	H8A—C8—H8B	108.6
C3—C4—C7	121.4 (3)	C9—C8—H8A	110.4
C5—C4—C7	119.2 (3)	C9—C8—H8B	110.4
C5—C4—C3	119.4 (3)	C8—C9—H9A	109.5
F1—C2—C1	116.7 (3)	C8—C9—H9B	109.5
F1—C2—C3	118.7 (3)	C8—C9—H9C	109.5
C3—C2—C1	124.7 (3)	H9A—C9—H9B	109.5
O2—C7—C4	111.9 (3)	H9A—C9—H9C	109.5
O1—C7—O2	123.9 (3)	H9B—C9—H9C	109.5
O1—C7—C4	124.2 (3)		
			
F1—C2—C3—C4	179.9 (3)	C7—C4—C5—C6	179.2 (3)
F2—C6—C1—N1	2.6 (5)	C3—C4—C7—O2	−0.8 (4)
F2—C6—C1—C2	−178.9 (3)	C3—C4—C7—O1	178.6 (3)
F2—C6—C5—C4	178.6 (3)	C3—C4—C5—C6	0.1 (5)
N1—C1—C2—F1	−1.7 (5)	C5—C6—C1—N1	−178.3 (3)
N1—C1—C2—C3	178.9 (3)	C5—C6—C1—C2	0.2 (5)
C6—C1—C2—F1	179.8 (3)	C5—C4—C7—O2	−179.9 (3)
C6—C1—C2—C3	0.4 (5)	C5—C4—C7—O1	−0.5 (5)
C1—C6—C5—C4	−0.4 (5)	C5—C4—C3—C2	0.4 (5)
C1—C2—C3—C4	−0.7 (5)	C8—O2—C7—O1	1.3 (4)
C7—O2—C8—C9	−176.7 (3)	C8—O2—C7—C4	−179.3 (3)
C7—C4—C3—C2	−178.7 (3)		

### Ethyl 4-amino-3,5-difluorobenzoate (II) Hydrogen-bond geometry (Å, º)

**Table:**

*D*—H···*A*	*D*—H	H···*A*	*D*···*A*	*D*—H···*A*
N1—H1*A*···O1^i^	0.85 (3)	2.15 (4)	2.942 (3)	155 (3)
C8—H8*B*···F1^ii^	0.99	2.46	3.015 (3)	115

Symmetry codes: (i) *x*, *y*−1, *z*; (ii) *x*, *y*+1, *z*.

### Diethyl 4,4'-(diazene-1,2-diyl)bis(3,5-difluorobenzoate) (III) Crystal data

**Table:**

C_18_H_14_F_4_N_2_O_4_	*Z* = 1
*M**_r_* = 398.31	*F*(000) = 204
Triclinic, *P*1	*D*_x_ = 1.536 Mg m^−^^3^
*a* = 4.6106 (17) Å	Mo *K*α radiation, λ = 0.71073 Å
*b* = 8.839 (3) Å	Cell parameters from 1029 reflections
*c* = 10.969 (4) Å	θ = 2.4–27.2°
α = 99.330 (8)°	µ = 0.14 mm^−^^1^
β = 99.431 (8)°	*T* = 100 K
γ = 96.442 (7)°	Needle, dark brown
*V* = 430.7 (3) Å^3^	0.57 × 0.13 × 0.1 mm

### Diethyl 4,4'-(diazene-1,2-diyl)bis(3,5-difluorobenzoate) (III) Data collection

**Table:**

Bruker SMART APEXII diffractometer	1913 independent reflections
Radiation source: sealed X-ray tube, EIGENMANN GmbH	1414 reflections with *I* > 2σ(*I*)
Graphite monochromator	*R*_int_ = 0.023
Detector resolution: 7.9 pixels mm^-1^	θ_max_ = 27.5°, θ_min_ = 1.9°
ω and φ scans	*h* = −5→5
Absorption correction: multi-scan (SADABS; Krause *et al.*, 2015)	*k* = −11→11
*T*_min_ = 0.926, *T*_max_ = 0.986	*l* = −12→14
3269 measured reflections	

### Diethyl 4,4'-(diazene-1,2-diyl)bis(3,5-difluorobenzoate) (III) Refinement

**Table:**

Refinement on *F*^2^	Primary atom site location: structure-invariant direct methods
Least-squares matrix: full	Hydrogen site location: mixed
*R*[*F*^2^ > 2σ(*F*^2^)] = 0.047	H atoms treated by a mixture of independent and constrained refinement
*wR*(*F*^2^) = 0.150	*w* = 1/[σ^2^(*F*_o_^2^) + (0.0945*P*)^2^] where *P* = (*F*_o_^2^ + 2*F*_c_^2^)/3
*S* = 1.01	(Δ/σ)_max_ < 0.001
1913 reflections	Δρ_max_ = 0.28 e Å^−^^3^
132 parameters	Δρ_min_ = −0.29 e Å^−^^3^
0 restraints	

### Diethyl 4,4'-(diazene-1,2-diyl)bis(3,5-difluorobenzoate) (III) Special details

**Table:**

Geometry. All esds (except the esd in the dihedral angle between two l.s. planes) are estimated using the full covariance matrix. The cell esds are taken into account individually in the estimation of esds in distances, angles and torsion angles; correlations between esds in cell parameters are only used when they are defined by crystal symmetry. An approximate (isotropic) treatment of cell esds is used for estimating esds involving l.s. planes.

### Diethyl 4,4'-(diazene-1,2-diyl)bis(3,5-difluorobenzoate) (III) Fractional atomic coordinates and isotropic or equivalent isotropic displacement parameters (Å^2^)

**Table:**

	*x*	*y*	*z*	*U*_iso_*/*U*_eq_	
F1	0.3292 (3)	0.83917 (13)	0.96263 (11)	0.0332 (3)	
F2	0.2359 (3)	0.43669 (13)	1.19462 (11)	0.0352 (3)	
O1	−0.2904 (3)	0.84303 (16)	1.39751 (13)	0.0308 (4)	
O2	−0.1813 (3)	1.04412 (15)	1.30209 (12)	0.0261 (3)	
N1	0.4466 (3)	0.55792 (18)	0.98505 (15)	0.0256 (4)	
C1	0.2996 (4)	0.6346 (2)	1.07526 (16)	0.0206 (4)	
C2	0.2364 (4)	0.7829 (2)	1.05820 (17)	0.0235 (4)	
C3	0.0879 (4)	0.8714 (2)	1.13506 (17)	0.0229 (4)	
C4	−0.0091 (4)	0.8109 (2)	1.23300 (17)	0.0212 (4)	
C5	0.0423 (4)	0.6636 (2)	1.25239 (17)	0.0232 (4)	
H5	−0.028030	0.621698	1.318322	0.028*	
C6	0.1961 (4)	0.5799 (2)	1.17490 (17)	0.0223 (4)	
C7	−0.1761 (4)	0.8990 (2)	1.32022 (17)	0.0223 (4)	
C8	−0.3390 (4)	1.1407 (2)	1.38246 (19)	0.0277 (4)	
H8A	−0.296123	1.121791	1.469698	0.033*	
H8B	−0.556011	1.116299	1.351234	0.033*	
C9	−0.2333 (5)	1.3059 (2)	1.3787 (2)	0.0347 (5)	
H9A	−0.274544	1.322754	1.291824	0.052*	
H9B	−0.018988	1.329046	1.411153	0.052*	
H9C	−0.337013	1.374039	1.430782	0.052*	
H3	0.060 (4)	0.969 (2)	1.117 (2)	0.027 (5)*	

### Diethyl 4,4'-(diazene-1,2-diyl)bis(3,5-difluorobenzoate) (III) Atomic displacement parameters (Å^2^)

**Table:**

	*U*^11^	*U*^22^	*U*^33^	*U*^12^	*U*^13^	*U*^23^
F1	0.0480 (7)	0.0331 (6)	0.0279 (7)	0.0169 (5)	0.0208 (5)	0.0111 (5)
F2	0.0462 (7)	0.0308 (6)	0.0397 (7)	0.0187 (5)	0.0220 (6)	0.0152 (5)
O1	0.0320 (7)	0.0348 (8)	0.0301 (8)	0.0085 (6)	0.0151 (6)	0.0072 (6)
O2	0.0271 (7)	0.0277 (7)	0.0256 (7)	0.0089 (5)	0.0108 (5)	0.0016 (6)
N1	0.0250 (8)	0.0295 (8)	0.0239 (8)	0.0092 (6)	0.0078 (6)	0.0028 (7)
C1	0.0174 (8)	0.0253 (9)	0.0183 (9)	0.0055 (6)	0.0029 (7)	0.0005 (7)
C2	0.0243 (9)	0.0293 (10)	0.0180 (9)	0.0056 (7)	0.0050 (7)	0.0056 (7)
C3	0.0246 (9)	0.0241 (9)	0.0210 (9)	0.0075 (7)	0.0039 (7)	0.0047 (7)
C4	0.0155 (8)	0.0270 (9)	0.0190 (9)	0.0031 (6)	0.0016 (7)	−0.0005 (7)
C5	0.0198 (8)	0.0297 (10)	0.0201 (9)	0.0036 (7)	0.0038 (7)	0.0042 (7)
C6	0.0214 (8)	0.0224 (9)	0.0233 (10)	0.0061 (7)	0.0018 (7)	0.0050 (7)
C7	0.0186 (8)	0.0279 (9)	0.0185 (9)	0.0036 (7)	0.0010 (7)	0.0010 (7)
C8	0.0258 (9)	0.0299 (10)	0.0283 (10)	0.0093 (7)	0.0117 (8)	−0.0026 (8)
C9	0.0398 (11)	0.0325 (11)	0.0329 (11)	0.0114 (9)	0.0109 (9)	0.0009 (9)

### Diethyl 4,4'-(diazene-1,2-diyl)bis(3,5-difluorobenzoate) (III) Geometric parameters (Å, º)

**Table:**

F1—C2	1.341 (2)	C3—H3	0.94 (2)
F2—C6	1.345 (2)	C4—C5	1.392 (3)
O1—C7	1.211 (2)	C4—C7	1.500 (2)
O2—C7	1.332 (2)	C5—H5	0.9500
O2—C8	1.463 (2)	C5—C6	1.373 (3)
N1—N1^i^	1.252 (3)	C8—H8A	0.9900
N1—C1	1.415 (2)	C8—H8B	0.9900
C1—C2	1.409 (3)	C8—C9	1.496 (3)
C1—C6	1.395 (3)	C9—H9A	0.9800
C2—C3	1.373 (3)	C9—H9B	0.9800
C3—C4	1.389 (3)	C9—H9C	0.9800
			
C7—O2—C8	116.47 (14)	F2—C6—C5	117.43 (16)
N1^i^—N1—C1	114.4 (2)	C5—C6—C1	122.86 (17)
C2—C1—N1	115.52 (16)	O1—C7—O2	124.89 (17)
C6—C1—N1	128.64 (16)	O1—C7—C4	123.19 (18)
C6—C1—C2	115.76 (16)	O2—C7—C4	111.91 (16)
F1—C2—C1	117.69 (16)	O2—C8—H8A	110.3
F1—C2—C3	119.19 (16)	O2—C8—H8B	110.3
C3—C2—C1	123.11 (17)	O2—C8—C9	107.28 (15)
C2—C3—C4	118.52 (17)	H8A—C8—H8B	108.5
C2—C3—H3	116.6 (13)	C9—C8—H8A	110.3
C4—C3—H3	124.9 (13)	C9—C8—H8B	110.3
C3—C4—C5	120.65 (17)	C8—C9—H9A	109.5
C3—C4—C7	121.92 (17)	C8—C9—H9B	109.5
C5—C4—C7	117.42 (17)	C8—C9—H9C	109.5
C4—C5—H5	120.5	H9A—C9—H9B	109.5
C6—C5—C4	119.07 (17)	H9A—C9—H9C	109.5
C6—C5—H5	120.5	H9B—C9—H9C	109.5
F2—C6—C1	119.66 (16)		
			
F1—C2—C3—C4	179.45 (15)	C3—C4—C7—O1	171.84 (16)
N1^i^—N1—C1—C2	166.37 (19)	C3—C4—C7—O2	−8.3 (2)
N1^i^—N1—C1—C6	−17.2 (3)	C4—C5—C6—F2	−178.54 (14)
N1—C1—C2—F1	−2.4 (2)	C4—C5—C6—C1	−1.2 (3)
N1—C1—C2—C3	178.22 (15)	C5—C4—C7—O1	−7.2 (3)
N1—C1—C6—F2	0.7 (3)	C5—C4—C7—O2	172.65 (14)
N1—C1—C6—C5	−176.52 (16)	C6—C1—C2—F1	−179.32 (14)
C1—C2—C3—C4	−1.1 (3)	C6—C1—C2—C3	1.3 (3)
C2—C1—C6—F2	177.21 (15)	C7—O2—C8—C9	161.06 (16)
C2—C1—C6—C5	−0.1 (3)	C7—C4—C5—C6	−179.59 (14)
C2—C3—C4—C5	−0.2 (3)	C8—O2—C7—O1	−0.2 (3)
C2—C3—C4—C7	−179.22 (14)	C8—O2—C7—C4	179.99 (13)
C3—C4—C5—C6	1.4 (3)		

Symmetry code: (i) −*x*+1, −*y*+1, −*z*+2.
